# Understanding Quality of Life for People with Motor Neurone Disease Who Use Tracheostomy Ventilation and Family Members: A Scoping Review

**DOI:** 10.3390/brainsci14080821

**Published:** 2024-08-16

**Authors:** Nicola Turner, Christina Faull, Jonathan Palmer, Alison Armstrong, Jennifer Bedford, Martin R. Turner, Eleanor Wilson

**Affiliations:** 1 School of Health Sciences, University of Nottingham, Nottingham NG7 2AH, UK; eleanor.wilson@nottingham.ac.uk; 2 LOROS Hospice Centre for Excellence, Leicester LE3 9QE, UK; christinafaull@loros.co.uk; 3 Department of Thoracic Medicine, University Hospitals Plymouth NHS Trust, Plymouth PL6 8DH, UK; jonathanpalmer@nhs.net; 4 North-East Assisted Ventilation Service, Newcastle upon Tyne Hospitals NHS Foundation Trust, Newcastle upon Tyne NE1 4LP, UK; alison.armstrong1@nhs.net; 5 Motor Neurone Disease Association, Northampton NN3 6BJ, UK; jennifer.bedford@mndassociation.org; 6 Nuffield Department of Clinical Neurosciences, University of Oxford, Oxford OX3 9DU, UK; martin.turner@ndcn.ox.ac.uk

**Keywords:** motor neurone disease, amyotrophic lateral sclerosis, tracheostomy ventilation, invasive ventilation, quality of life, wellbeing, family care

## Abstract

Tracheostomy ventilation (TV) can increase survival time for people living with motor neurone disease (MND); however, the use of TV varies between countries. Concerns regarding anticipated quality of life (QoL) are among the reasons given by healthcare professionals for not recommending this intervention, yet little is known about QoL in this context. This scoping review was conducted to examine the evidence on QoL for those with MND who use TV and family members involved in their care. Using the methodological guidance of the Joanna Briggs Institute, 23 papers were identified for inclusion, and findings were inductively analysed to identify key themes. We found that people living with MND tend to rate QoL post TV more positively than anticipated by healthcare professionals or family members. QoL was found to be related to positive relationships and activities the person could maintain. Feeling able to make a choice and an adequate level of financial resources were also important factors. Family members tended to experience lower QoL, associated with the uncertainty surrounding an emergency procedure and the complexity of subsequently required care. More evidence on QoL from the perspectives of people with MND who use TV is needed to support decision making and inform guidance.

## 1. Introduction

Motor neurone diseases (MNDs) are a rare group of progressive, neurological conditions characterised by degeneration of the motor neurones. Amyotrophic lateral sclerosis is the most common form, presenting with a combination of upper and lower motor degeneration. Less common variants include primary lateral sclerosis and progressive bulbar palsy (PBP), with progressive muscular atrophy (PMA) affecting very few people [1]. In the UK, the overarching term MND is used for these conditions and is used throughout this paper.

Published guidance in Europe recommends non-invasive ventilation (NIV) for the management of respiratory insufficiency for people living with MND (plwMND), with tracheostomy or invasive ventilation (TV) more likely to be considered an option in circumstances where NIV is inadequate [2]. There is no reference to TV in the UK guidance on the management of MND [3].

Rates of use of TV for plwMND vary between countries. For example, in Japan, 29–38% of plwMND receive TV, while in mainland Europe and the United States, TV use varies from approximately 5% to 10% [4,5,6]. In the UK, only 1% of plwMND use TV, with 81% of such uses being placed in an emergency [7]. The organisation of funding for health and care provision in different health care systems may contribute to the variation in TV rates [8]. A high level of complex care in the community is required for people to be accommodated at home following the initiation of TV. This may not be readily available or be seen as cost-effective, especially given the limited data on quality of life (QoL) for plwMND who use TV, as well as family members involved in their care.

There is evidence that TV can increase survival time for plwMND when compared with NIV [9,10]. A longitudinal case–control study reported a difference of almost 7 years in life expectancy between Japanese MND patients who did and did not receive TV [4]. It is known that men—especially younger men—are twice as likely as women to be affected by MND [11] and seem more likely to choose invasive treatment to prolong life [7,12,13]. This may, in part, be due to the availability of care from a family member [14].

Evidence suggests that continuous invasive ventilation has variable effects on health-related QoL and can be dependent on diagnosis and comorbidities. For example, those with lung disease, such as COPD, are more likely to be older and have comorbidities contributing to worse QoL scores when compared to those with slowly progressive neuromuscular disorders [15,16,17]. Concerns related to the impact of TV on QoL for plwMND and their family members are often cited by healthcare professionals as reasons for not recommending TV for plwMND. These concerns include potentially long inpatient hospital stays, the detrimental impact of the procedure on the person’s ability to communicate, and the risk of becoming locked in [18,19]. However, few studies have set out to investigate QoL from the perspectives of plwMND who use TV themselves [20], and there has been little qualitative exploration of what QoL means for people in this context.

The impact of TV on QoL for plwMND and their family members is not well understood. This scoping review aims to locate the available evidence and examine how QoL has been conceptualised and measured in the research literature to date [21]. This is part of an ongoing qualitative study funded by the UK Motor Neurone Disease Association to explore living with TV in MND and its impact on QoL, the protocol of which is published elsewhere [22]. This foundation is essential for identifying gaps in the evidence base necessary to inform future research, policy, and practice. A better understanding of the impact of TV on QoL will enable plwMND, family members, and healthcare professionals to make informed decisions regarding future care. This scoping review follows the guidance of the Joanna Briggs Institute (JBI) [23] to identify and describe the evidence currently available. The aim was to explore what is known about quality of life for (a) those with MND who use tracheostomy ventilation to support their breathing and (b) family members involved in their care.

## 2. Methods

A scoping review aims to map the scope of the current literature in a particular area and is particularly useful for examining complex or emerging topics, identifying gaps in the literature, and informing the development of future research agendas. Unlike systematic reviews that focus on answering specific research questions with a predefined set of criteria, scoping reviews are broader in scope, seeking to identify and summarise the extent and nature of existing evidence [21]. This scoping review applied the Joanne Briggs Institute framework to locate the available evidence and describe concepts and contextual information relevant to understanding QoL for plwMND who use TV and family members involved in their care [23]. The Preferred Reporting Items for Systemic Reviews and Meta-Analyses Extension for Scoping Reviews (PRISMA-ScR) was used to guide the reporting of this review [24]. The scoping review protocol is available via the Open Science Framework at https://doi.org/10.17605/OSF.IO/4P8AM (accessed on 27 June 2024).

### 2.1. Inclusion and Exclusion Criteria

The question and inclusion criteria for the scoping review were formulated using the recommended framework to identify the population, concept, and context that provide the focus for the review. Inclusion and exclusion criteria are reported in Table 1.

### 2.2. Evidence Sources

The scoping review included empirical studies of any design using quantitative, qualitative, or mixed methodologies. Relevant case reports, reviews, and pre-print publications were also considered for inclusion. The inclusion and exclusion criteria for evidence sources are summarised in Table 1.

### 2.3. Search Strategy

An initial search of Medline via OVID and CINAHL via EBSCO was undertaken to identify articles on the review topic. Keywords contained in titles and abstracts of relevant articles and index terms applied to articles were used to further develop and adapt the search strategy for use with other databases. Additional searches were carried out in the AMED, Books@Ovid, The Cochrane Library, EMBASE, and PubMed Central databases. Initial search terms are presented in Box 1.

Box 1 **Initial search terms.**

MND/or ‘motor neurone disease’/or ALS/or ‘amyotrophic lateral sclerosis’/or ‘Lou Gehrig’s disease’

AND

TV/or ‘tracheostomy ventilation’/or IV/or ‘invasive ventilation’

AND

‘quality of life’/or QUOL/or ‘health related quality of life’/or HQOL/or wellbeing/or burden

Searches were limited by applying the categorical terms human adults (aged 18 and above) to publications where the full text was available and could be accessed by the research team in English. Evidence published from 2000 onwards was included; the rationale for excluding evidence prior to this date relates to advances to procedures surrounding the initiation of TV and post-operative support, which are likely to influence QoL for plwMND and their family members and, therefore, to make the findings of earlier research less relevant to building a contemporary understanding.

### 2.4. Screening and Study Selection

Citations identified during the search phase were uploaded into EndNote X9© (Clarivate, Philadelphia, PA, USA) for storage and retrieval. Following the removal of duplicates, 429 papers were identified for initial screening. Papers were excluded at this stage if they did not include information on QoL for plwMND who use TV. Sources such as conference abstracts and correspondence were also excluded. Titles and abstracts were screened against the inclusion criteria by N.T. and 38 records were retained for full-text review.

**Figure 1:**
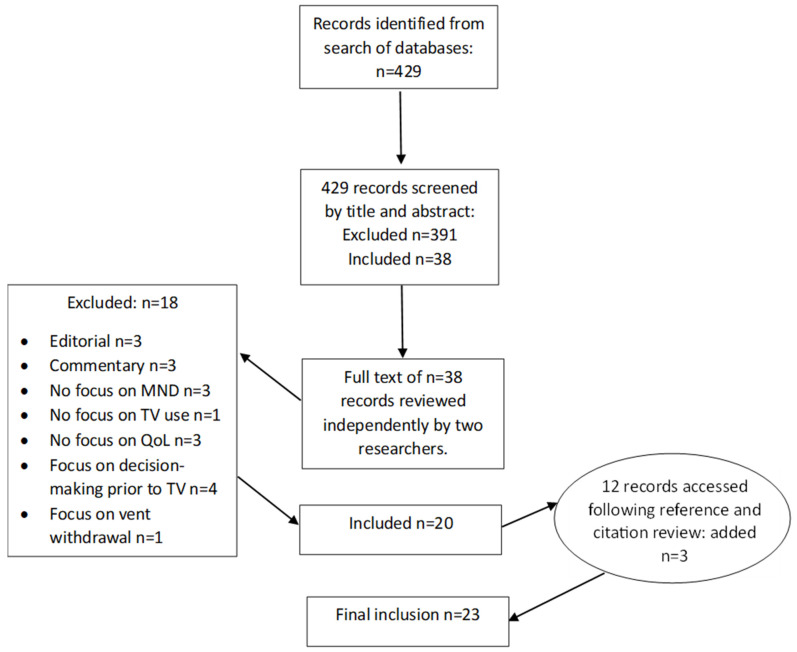
PRISMA flow chart of literature screening process.

A full-text review of the 38 papers was carried out independently by N.T. and E.W., and notes were collated in a shared file to enable comparison. Any difference in interpretation of relevance between reviewers was resolved through discussion and by referring back to the source until agreement was achieved. This phase resulted in the selection of 20 papers for inclusion. Reasons for the exclusion of full-text sources were recorded and are reported in the PRISMA-ScR flow diagram [25] presented in Figure 1.

The reference lists and citations of the 20 papers were reviewed by N.T., resulting in 12 additional papers being accessed. Of these, 3 were considered relevant for inclusion, leading to a final total of 23 papers for extraction of relevant information and charting.

### 2.5. Extraction and Charting 

The 23 papers selected for inclusion were divided between five members of the review team (N.T., E.W., C.F., M.T., and J.B.) for independent extraction of relevant information using a spreadsheet developed by N.T. in consultation with team members. This was used to collate specific details about the participants, context, study methods, and key findings relating to QoL for plwMND who use TV and/or family members. Critical appraisal of evidence sources is not essential for scoping reviews and was not undertaken, although any limitations for the purpose of the review were noted. The completed table charting all included papers is available in the Supplementary Materials (Table S1). Once familiar with the included articles, N.T. and E.W. individually identified key aspects of the study findings and attributed descriptive codes in relation to the review aim [23]. Codes were compared, refined, and organised into key categories pertaining to QoL for plwMND and family members. Articles used terms ‘next of kin’, ‘caregiver’, and ‘carer’ to denote family members with unpaid caregiving responsibilities for the plwMND. We use the overarching term ‘family member’ throughout this article for consistency.

## 3. Findings

The scoping review included three previous review papers relevant to understanding current evidence on QoL for plwMND who use TV or their family members. One recent non-systematic review presented a summary of the literature on the use of several interventions by plwMND, including TV, focussing on the impact on progression of MND and survival rates [18]. In a brief section addressing QoL for plwMND following TV, the authors concluded that “there is complete agreement in the literature that no significant deterioration in QoL occurs”. A systematic review of the suitability of TV for plwMND [19] reported that those who use TV are more likely to be satisfied with their QoL when living at home; however, for family members involved in their care, a substantial negative impact was associated with managing TV in the home. Lulé et al. [26] provided a non-systematic review of the literature on patient adjustment to locked-in syndrome. For plwMND, social support was identified as crucial for maintaining QoL. However, the rationale for study selection was not provided, and two of the four studies involving plwMND were unpublished.

**Figure 2:**
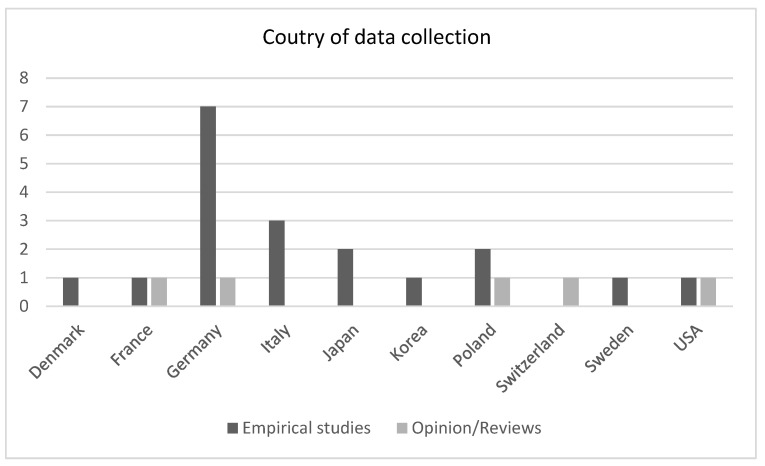
County of data collection. Two studies collected data across two countries (Barras et al., 2013, Switzerland and France [19]; Barć et al., 2022, Poland and Germany [28]).

The remaining twenty papers included in this scoping review were empirical studies using a range of methods to explore QoL in relation to TV use from the perspectives of plwMND, family members, or healthcare practitioners. Further information on study participants and methodologies is provided in Table 2, and information regarding country of origin is presented in Figure 2. Dates of publication were between 2003 and 2023, with 16 of the 23 (70%) publications appearing in the last 10 years.

Evidence of healthcare professionals’ views on QoL for plwMND who use TV was drawn from two linked studies using bespoke postal or online surveys completed by neurologists in Germany [27] and in Germany and Poland [28]. Both studies used a ten-point Likert scale to report on neurologists’ assessments of QoL and depressiveness of plwMND who use either NIV or TV and/or nutrition via percutaneous endoscopic gastronomy (PEG). Aho-Özhan et al. [27] found that more experienced neurologists were better able to estimate a QoL score closer to the subjective assessment of 52 plwMND using one or more of these therapeutic interventions derived from a previous study [29]. Barć et al. [28] reported that demographic factors and cultural background can affect a neurologist’s assessment of QoL, in addition to experience. Aho-Özhan et al. [27] acknowledged that self-reported well-being varies greatly between individual plwMND who have received the same intervention and that a limitation of this approach is that it does not compare neurologist and plwMND ratings of QoL for specific cases. Furthermore, no analysis was conducted with respect to how the concept of QoL was interpreted by each group, and no exploration of the views of other healthcare professionals involved in the care of plwMND was reported.

The remaining eighteen studies examined the perspectives of plwMND who use TV and/or the perspectives of family members involved in their care. Eleven studies applied a range of validated questionnaires to assess QoL for plwMND and family members, together with measurement of associated variables such as depression and anxiety, coping, and the burden associated with providing care (see Table 3). In a further two studies, a bespoke questionnaire was devised. Five studies adopted qualitative methods to explore QoL; four involved interviews with family members only, and one consisted of a single narrative account of a plwMND’s experience of using TV. In the following section, we summarise key aspects of QoL for plwMND who use TV and family members as described in the findings of these studies.

### 3.1. Quality of Life: People Living with MND

The literature highlights that QoL is often rated as good by plwMND who use TV themselves. Table 3 shows the range of QoL measures used in the reviewed studies. PlwMND report high levels of optimism and enjoyment [30] and generally maintain a positive perspective [31,32], even when completely immobile and unable to communicate verbally [33]. Rabkin et al.’s [30] longitudinal study of 14 plwMND who chose TV went on to show some decline in levels of optimism and enjoyment after an average of 33 months post procedure. They noted the need to recognise that plwMND appreciate that life is worth living but that as time moves on, their subjective assessments of what constitutes a worthwhile life may change.

Kaub-Wittemer et al. [34] and Rousseau et al. [35] both reported that QoL for plwMND who use TV is no different than that for those using NIV. As part of a wider study of survival and QoL after tracheostomy for acute respiratory failure, Vianello [31] administered QoL questionnaires to 13 plwMND one month post discharge from hospital. Findings showed that when compared to a control group of plwMND who did not use mechanical ventilation, there were no significant effects of commencing the use of TV on any of the QoL domains. However, Beck Depression Inventory scores identified that a small proportion (*n* = 2/13 or 15%) of the participants were severely depressed. In this small sample, it is not possible to determine whether depression was related to the initiation of TV or to other factors.

Several studies have explored different aspects of QoL to clarify why plwMND may self-rate QoL higher than that expected by healthcare professionals or family members [17,26,29,33]. In these papers, QoL was not found to be linked to physical functioning; those with TV positively rated aspects of their lives that were less impacted by disease, such as family, marriage, and friends [20]. Hirano et al.’s [36] study of Japanese plwMND identified that more sources of psychosocial support and happiness were associated with greater hope. Over 90% of participants identified at least one source of happiness, with enjoyment of television/radio/music/nature, the presence of children/grandchildren, bathing, and human interactions/conversation rated most highly. In Sutherland’s [37] personal narrative of his decision to embrace TV, he highlighted the importance of being able to maintain dignity by contributing to social relationships:

*I have learned I can maintain my dignity by caring for people in other ways. Through being kind, appreciative, listening, and supportive, I have found great self-worth.*

[37] (p. 281)

Whether TV was chosen by the plwMND or placed in an emergency without consent is considered a factor that could impact QoL. Those actively choosing to have TV were identified as having a “*more positive appraisal of their ability of function in daily life, their physical health and overall life satisfaction*” [30] (p. 86). They were also more likely to be male, have younger children, have a higher level of education, and be from higher-income households [30,34]. Sutherland [37] discussed his financial position as a consideration in his decision to undergo TV, and Veronese et al. [38] noted that the ability to access paid help once TV is in place supports QoL. Indeed, Hirano et al. [36] found that in Japan, higher economic status correlated with lower levels of emotional and physical challenges, illustrating a positive correlation in plwMND between identifying multiple sources of happiness and increasing economic status. This was assessed in terms of being able to afford common amenities and services, with the authors suggesting a link with direct care limitations and concerns of being an additional financial burden for families.

For those who initiated TV in an emergency, evidence from multiple studies showed that despite the challenges, the majority of plwMND would choose TV again [20,31,33,34]. However, there may be contextual and cultural variations; Veronese et al.’s [38] qualitative study conducted in Italy, where Advanced Decisions to Refuse Treatment (ADRTs) are not legally binding, suggested that pressure from healthcare professionals, having little time to decide, and ADRTs being overridden had a negative impact on QoL. Drawing on proxy accounts from family members, the authors reported that QoL worsened for half of the plwMND in their study. In addition to feelings of having been coerced by healthcare professionals, this was attributed to the continued progression of the disease and disintegrating hope of recovery. PlwMND also self-reported concerns in Hirano et al.’s [36] questionnaire study—most frequently, difficulties with communicating post TV, followed by fear of the burden on family members, fear of losing all mobility, and fear of mechanical failure of home ventilation equipment. Two other included articles cited fear of being a burden on others as a predictor of not choosing TV [29,37]. Clinical impacts such as dyspnoea and hypoventilation, which resulted in more frequent hospital admissions [39], and longer recovery times in hospital following placement of TV in an emergency also impacted negatively on QoL for plwMND [19].

### 3.2. Quality of Life: Family Members

Evidence from studies identified in our review suggests that QoL for family members is lower than that of plwMND who use TV [18,20,34]. Findings from Tülek et al.’s Turkish study [40] correlate higher caregiver burden with higher levels of anxiety and depression, less social support, and subsequently worse QoL. According to their findings, 52% of family caregivers were anxious, and 58% were depressed. However, in a recent German cross-sectional observation study, the authors reported that over half of plwMND but only 4% of family members were using antidepressants [20].

Several studies included in the review explored the nature of the burden on family caregivers in greater depth [30,34,41,42,43,44]. Gottberg et al. [41] reported that the emergency placement of TV could be chaotic and frightening for family members. This qualitative interview study with eight family members in Sweden found that while TV had often been discussed in advance, the decision to initiate was eventually made in an emergency, where family members experienced high levels of uncertainty. Decision making was described in the context of “*the uncertain prognosis when [TV] was initiated, whether the patient would survive surgery, and how the near future would be…*” [41] (p. 2405).

As evidenced in Veronese et al.’s [38] Italian study described above, decisions to start TV were often rushed, and family members—similar to plwMND—felt insufficiently informed. If plwMND had chosen TV for themselves, family members expressed less hesitancy and did not regret the decision afterwards [42]. For those included in Akiyama et al.’s [42] qualitative study conducted in Japan, the need to find meaning in the prolongation of the plwMND’s life was an important facet of family members’ QoL. Family members reported being able to find ‘meaning in prolonging life’ (p. 39) when they were supported by those around them. Without this care and support, family members felt they had to sacrifice their own life to provide care. Kaub-Wittemer et al. [34] reported that when comparing NIV with TV, 60% of family caregivers of plwMND who use TV had given up paid work, compared to 19% of family caregivers in the NIV group, although it is noted that only 6% of those in the NIV group were using mechanical ventilation for 24 h per day, compared to 95% in the TV group. Complexity of care was reported to be higher for family members in relation to TV use [44], with extensive physical and emotional burden and fear of burnout [30]. In the follow-up phase of Rabkin et al.’s [30] study, only 6 of the 13 caregivers remained living with the plwMND; one plwMND had died, but others were living in institutional settings or living alone with paid homecare workers employed to support their use of TV.

Once the plwMND was discharged from hospital and living at home, family members reported experiencing a lack of boundaries and potential conflicting roles as both a relative and a carer [41]. Like those in Akiyama et al.’s study [42], family members in Gottberg et al.’s study [41] described putting their ‘own life on hold’:

*It is as if my life was at a standstill because of the way the situation is. One works, eats, sleeps; that is evidently all one has time for.*

Next of kin 5 [41] (p. 2405)

Family members described daily routines transformed to focus on maintaining the plwMND’s well-being, which remained a priority regardless of their involvement in everyday care. Family members reported having to act as care co-ordinators and being permanently on standby to step in during emergencies [41]. These findings echo those of Winther et al. [43], who undertook interviews with nine family members of plwMND using TV in Denmark. Their thematic analysis described key aspects of the care burden experienced by family members, including the complexity of care when being required to act as care co-ordinators, paid homecare workers as an essential support but also an additional burden, living on permanent standby, and needing personal space. Those sharing the same home as the plwMND and lacking space for a personal and social life had higher levels of burden [40,41,43]. The home environment was described as having been transformed into a clinical space, with equipment, constant noise from machines, and ever-present homecare workers in attendance [41].

Paid homecare workers are recognised as playing an essential role in the care of plwMND who use TV [43]. However, family members noted that it could be difficult to find suitable homecare workers, with family members as the inevitable fall-back option when no homecare support is available [41]. Furthermore, a lack of trust in the knowledge, skills, and abilities of homecare workers could make it difficult for family members to leave them in charge, preventing family members from feeling able to take a break [43]. It is interesting to note that there was no information on the involvement of homecare workers as a factor related to QoL from the perspectives of plwMND who use TV.

A concern shared by plwMND and family members is the loss of communication associated with advanced MND and TV [36,41]. Family members noted how challenging communication could be once the plwMND was unable to articulate their needs clearly. The skill required to communicate with different people, including homecare workers, was also highlighted [41].

Some positive aspects of TV use were identified by family members. Some reported that once TV was in place, the increased involvement of homecare workers and more structured training on managing TV at home were beneficial [41]. Tülek et al. [40] noted that there were no differences in caregiver burden between patients with and without respiratory, speech, and feeding problems, suggesting that it may not be the introduction of artificial ventilation alone that creates such burdens of care. There was also some recognition of satisfaction with caregiving; in Winther et al.’s [43] study, family members described being ‘in this together’ and living with TV as a ‘joint enterprise’. Maintaining the QoL of the plwMND was perceived as an important part of the family members’ role [43]. In spite of the greater burdens, the TV group in Rabkin et al.’s study [30] also experienced more caregiver satisfaction than those in the NIV study group.

## 4. Discussion

This scoping review has drawn together evidence that suggests plwMND who use TV tend to rate their QoL more positively than might be anticipated by healthcare professionals or family members [26,27]. However, the tendency to positively rate QoL may be less likely when TV is placed in an emergency or when plwMND feel under pressure to proceed with the intervention. This is consistent with the argument that it is the opportunity to make a choice rather than the choice itself that determines satisfaction in the healthcare context [45].

It appears that plwMND who use TV may be more disposed to define QoL in terms of what they can do and enjoy rather than what they are no longer able to do due to the limitations imposed by their condition. This suggests that the process of adjustment to living with MND may facilitate a reduction in the gap between hopes and expectations hypothesised by Calman [46] as a measure of QoL. According to Calman, the narrower the gap, the more likely a person is to describe their QoL as positive at that time.

The reviewed evidence suggests being able to maintain social relationships with family and friends is important to QoL, along with adequate support for delivering complex care and managing equipment in the home. Financial stability was illustrated to be an important factor in determining QoL when living with TV [30,34,37]. This can impact the provision of paid homecare, medical expenses, and overall living conditions and is an important contributor to wider health inequalities, even in countries with healthcare systems that are free at the point of access, such as the UK [30,36,47].

The evidence compiled in this review highlights substantial impacts for family members who co-ordinate and provide the care that enables plwMND to retain their social connections and overall well-being. QoL for family members is influenced by the growing demands on their time and resources and the subsequent limitations on what they can do for themselves. Nevertheless, some family members report deriving satisfaction from their caring role. It may be of benefit for future research and interventions to focus on how this satisfaction may be enhanced, as well as how the outlined burdens could be reduced. Experiences of and attitudes towards providing care for plwMND are highly likely to be culturally specific, influenced by expectations of the role of family members, especially women, and the social care policies that determine the availability of paid care [48,49]. The ongoing uncertainty associated with living with a progressive, terminal illness is a factor affecting QoL for both plwMND and family members and is not exclusive to experiences of TV [50].

This scoping review has identified several gaps in our understanding of living with TV in MND. With the exception of a single, individual account [37], we were unable to identify any qualitative studies where a more subjective definition and interpretation of QoL was explored from the perspective of plwMND who use TV. Included studies rarely provided details of when in the post-tracheostomy trajectory they were carried out. Perceptions of QoL for plwMND and family members may change over time, and studies exploring QoL in the immediate, medium and longer term post tracheostomy would be of benefit. Given the low rate of TV use among people with MND in the UK, it is perhaps not surprising that none of the studies were undertaken in a UK context. In some included studies, only a small subset of the overall sample consisted of plwMND using TV [29,32]. Further research focusing on QoL for plwMND who use TV would contribute a better understanding of the specific QoL issues for this group. To date, we continue to have little understanding of how particular demographics, disease stages, geographic locations, or changes over time affect QoL for plwMND or their family members. While financial resources were identified as beneficial, specific costs were not explored. This field would benefit from further research and reviews to highlight the evidence on the wider costs of TV-related care over time, which vary across—and even within—countries.

This review highlights the importance of paid homecare workers with respect to the QoL of family members, yet little is known about the roles and experiences of this workforce in delivering care [51], especially in the context of caring for plwMND who use TV. There is some evidence from the UK that a lack of suitable care provision at home affects decisions to offer TV and significantly delays discharge after the procedure [8]. A breakdown in care, compounded by inadequacies in homecare services, can also result in unwanted hospital or hospice admissions [52]. Further exploration of homecare worker roles, responsibilities, and relationships within the family dynamic is important to improve care and support for plwMND and their family members when complex interventions, such as TV, are in place.

### Limitations of the Review

This review used search terms for MND and ALS but did not specify further subtypes of this disease group. Within the reviewed articles, there was variability in whether or not other motor neurone diseases were included in their samples. This inconsistency may impact the application of the findings across this group of conditions. Understandings of QoL in the included studies are based on varying conceptualisations of ‘quality of life’, often derived using standardised and/or non-standardised scales. Some were developed specifically for use with plwMND; others are generic scales that may not readily transfer to people with a serious, progressive illness. For family members, QoL was sometimes investigated using measures of carer burden, which may be a valid proxy for QoL or may result in findings that are skewed towards a more negative interpretation. Furthermore, different methods of administering scales were applied; some studies adapted questionnaires for use with eye-gaze technology, whilst others relied on family members to provide responses for plwMND or did not specify how the measure was completed. Heterogeneity in the use and application of measurement tools may affect cross-comparison between studies, with implications for the conclusions drawn. Furthermore, it is possible that the findings of studies included in the review may reflect an underlying tendency for plwMND who elect to use TV to be more positive in outlook and more determined to live longer, regardless of the intervention under review. Those who experience positive outcomes from TV may also be more inclined to participate in research.

Methodological limitations include the challenge of ensuring that all relevant literature was accessed. ‘Quality of life’ is a complex and multi-faceted concept. It is possible that the use of additional search terms such as ‘life satisfaction’ may have yielded more papers for inclusion. Limiting the search to papers where the full text was available in English may have also led to the exclusion of relevant material from countries where rates of TV for plwMND are higher.

Owing to its nature, a scoping review provides a broad overview of the available literature and does not involve quality assessment. This review considered a specific area of deliberately limited scope; therefore, studies with inconsistent and unclear methodologies were included in order to identify as much of the relevant information as possible. This has an impact on conclusions and recommendations.

## 5. Conclusions

TV is a relatively uncommon intervention for plwMND in comparison to NIV, and there is markedly less research literature on the experiences of plwMND who use TV to inform understanding. This review sought to draw together the small body of evidence that encapsulates QoL data from the perspectives of plwMND and their families. A better understanding of both the positive and negative impacts of TV on QoL may enable plwMND and family members to be better informed and prepared. This review highlights key differences between the perceptions and experiences of QoL of plwMND and family members. This is essential knowledge to support the process of informed decision making, especially when family members play such an important role in providing care that can enhance QoL for plwMND. While it is outside the scope of this review to make recommendations for clinical practice, we noted several important areas for further research to ensure that evidence is available to inform guidance and support practice in the future.

## Figures and Tables

**Table 1:** Inclusion and exclusion criteria.

Criterion	Inclusion	Exclusion
Population	PlwMND who use TV Family members with experience of supporting someone with MND to use TV Health and care professionals with experience working with someone with MND who uses TV	Does not report on plwMND who use TV or their family members
Concept	QoL QoL measures Benefits/challenges of using TV Burden	Does not report on QoL of plwMND who use TV or of their family members
Context	Home or usual place of residence Health and social care settings International	
**Evidence Sources**		
Study design	Experimental and quasi-experimental trials, observational studies, case studies, qualitative descriptive studies, and reviews	
Publication type	Peer-reviewed publications, pre-prints, textbook chapters, case reports, and full-text conference proceedings	Unpublished research, editorials, commentaries, conference abstracts, correspondence, policy, and guidance documents
Language	Full-text available in English	

**Table 2:** Study participants and methods.

Participants	No. of Studies	Method for Measuring QoL	Number of Participants
HCPs	2	Bespoke survey	570 (neurologists)
plwMND	7	Validated questionnaire	555 (78 TV)
	1	Single case narrative	1
Family members	4	Qualitative interview	50 (46 TV)
	1	Bespoke questionnaire	70 (24 TV)
plwMND + family members	4	Standardised questionnaire	317 plwMND (147 TV) 156 FM (37 TV) *
	1	Bespoke questionnaire	157 dyads (157 TV)

* 1 paper did not specify the number of plwMND using TV.

**Table 3:** Quality of Life (QoL) and related measures in included studies.

QoL measures (plwMND and family members)
Amyotrophic Lateral Sclerosis Assessment Questionnaire (ALSAQ) Anamnestic Comparative Self-Assessment (ACSA) European Quality of Life-Five Dimensions Questionnaire (EQ-5D) Life Satisfaction Index (LSI-11) McGill Quality of Life Single-Item Scale (McGill-SIS) Munich Quality of Life Dimensions List (MLDL) Quality of Life Enjoyment and Satisfaction Questionnaire (Q-LES-Q) Schedule for the Evaluation of Individual Quality of Life (SeiQoL) Schedule for the Evaluation of Individual Quality of Life—Direct Weighting (SeiQoL-DW) Severe Respiratory Insufficiency Questionnaire (SRI) Short Form 36 (SF-36)
Measures of depression and anxiety (plwMND and family members)
Allgemeine Depressionskala (ADSK) ALS Depression Inventory—12 items (ADI-12) Beck Depression Inventory-II (BDI-II) Centre for Epidemiologic Studies Depression Scale (German version) (CES–D) Hospital Depression and Anxiety Scale (HADS)
Other measures related to QoL (plwMND)
Motor Neurone Disease Coping Scale Schedule of Attitudes Toward Hastened Death (SAHD)
Other measures related to QoL (family members)
Burden Scale for Family Caregivers, short version (BSFC-s) Profile of Mood States (POMS) Zarit Burden Interview (ZBI)

## Supplementary Materials

The following supporting information can be downloaded at: https://www.mdpi.com/article/10.3390/brainsci14080821/s1, Table S1: Charting of included papers.
