# Supplementary material for: Understanding Quality of Life for People with Motor Neurone Disease Who Use Tracheostomy Ventilation and Family Members: A Scoping Review
Source: Brain Sci. 2024 Aug 16;14(8):821. doi: 10.3390/brainsci14080821 (PMC11352738; doi:10.3390/brainsci14080821)
Supplement: Supplementary file 1 [file brainsci-14-00821-s001.zip › brainsci-3097673-supplementary.pdf]

Table S1: TVLife Scoping Review charting of papers for inclusion

Author/Date/Title	Country	Study Design/ Method	Participants	Findings relevant to understanding QoL for PlwMND who use TV	Limitations for the purpose of the review
Aho-Ozhan, H. et al. (2017). Experience matters: neurologists' perspectives on ALS patients' well-being. [27]	Germany	Questionnaire	Neurologists n=105	Life-prolonging measure were estimated to result in increased patient depressiveness. Regarded TV as measure associated with higher depression and lower QOL. Those with palliative care training estimated higher QoL for PEG and NIV but not for TV.	Reports neurologists' general perspectives on QoL, not for specific plwMND. Acknowledges that significant variation in psychological wellbeing of plwMND would be anticipated.
Akiyama MO, Kayama M, Takamura S, et al. (2006) A study of the burden of caring for patients with amyotrophic lateral sclerosis (MND) in Japan. [42]	Japan	Qualitative interview study using grounded theory	12 family members of plwMND (10 TV, 2 NIV).	Family members tried to 'find a meaning in prolonging life,' (core category). Two subcategories:<br>1) hesitation and regret over the decision<br>2) feeling of being supported.	NIV/TV findings not reported separately.<br>Feelings of being supported likely to be influenced by positive relationship with key physician. Since recruitment was via physician, those with less positive relationship may not have been included.
Aust, E et al. (2022). Quality of life and mental health in the locked-in-state-differences between patients with amyotrophic lateral sclerosis and their next of kin. [20]	Germany	Cross-sectional observation study using a battery of questionnaires and additional quantitative interviews with next of kin.	PlwMND (n=15) in an incomplete locked-in-state and their next of kin	On the group level next of kin assessment of patient QoL did not differ from the patient's assessment of their own QoL – (but when examining them as pair there were 'considerable misjudgements', underestimating patient's QoL – evidence inconsistent). Patients over estimated next of kin's QoL. Next of kin's self-rating of Anxiety and Depression were both higher than patients. Patient most strongly rate the aspects of life not so impacted by their	Only includes those already in a LIS. 11/15 included were on TV and not possible isolate results for these

				disease. Despite most TVs placed in emergency all would choose this again.	
Barc, K., & Kuzma-Kozakiewicz, M. (2020). Gastrostomy and mechanical ventilation in amyotrophic lateral sclerosis: How best to support the decision-making process? [18]	Poland	Review of literature. No methodology cited.	Nutrition and ventilation papers	Focus on PEG, NIV and TV. Predictors of favourable attitude towards the use of TV are younger age, having young children, use of PEG and higher income and previous use of NIV.<br>Identifies agreement in the literature that TV causes no significant deterioration in QoL	A review not primary data. Summary rather than a literature review.<br>TV only one section and QoL only a section of that.
Barc, K., et al. (2022). Well-being on supportive techniques in amyotrophic lateral sclerosis: from neurologists' perspective. [28]	Poland & Germany	Questionnaire sent out by post and email on perceptions of QoL for ALS patients in relation to interventions including NIV and TV	Neurologists (n=465)	Overall perceived low QoL for TV (neutral for NIV).<br>Greater experience and palliative care training improved perceptions of QoL and reduced Depression score perception for TV and NIV respectively.<br>Higher estimations of depressiveness when discussions about intervention were later in disease course.	Reports neurologists' general perspectives on QoL, not for specific plwMND. Overall analysed response rate 15%
Barras, A. et al. (2013). Is tracheostomy still an option in amyotrophic lateral sclerosis? Reflections of a multidisciplinary workgroup. [19]	Switzerland and France	Systematic review of literature (In French and English and inc. only clinical trials) Discussion with specialist MND centres, home care societies and palliative acre wards.		Often TV initiated in an emergency following respiratory failure – and non-elective.<br>QOL is affected (literature and according to Swiss and French clinics) adversely if the patient is institutionalised after TV. The ability to return home is an important consideration in the decision for MND patients.	Limited reporting of review strategy and outcomes. Six of the eight tabled papers were published in, or before, 2010.
Gottberg, K., et al. (2021). Experiences of next of kin to patients with amyotrophic	Sweden	Semi-structured interviews.	Family members (n=8)	3 main themes:<br>1) All initiated as an emergency – 'chaotic and frightening'	Limited sample as 3/8 next of kin related to one plwMND.<br>All TV placed in an emergency

lateral sclerosis using invasive ventilation via tracheostomy. [41]		Qualitative content analysis	identified by plwMND	2) Struggling to cope with the strains of everyday life<br>3) Conflicting roles as next of kin and carer, lack of boundary been formal and informal care.	
Hirano, Y. et al. (2006). Ventilator dependence and expressions of need: a study of patients with amyotrophic lateral sclerosis in Japan. [36]	Japan	Interview to inform a questionnaire	plwMND (n=27) and family members (n=27)<br>Survey (n=157) plwMND only	Family member were full care providers, 45% were aged 60+. 30% place in emergency without patient or family member consent.<br>Concerns due to difficulty communicating, fear of the burden of care on family member health, fear of losing all mobility, mechanical failure of vent. 65% also experience concerns about financial burden. Higher economic status correlated with lower emotional and physical difficulties, living at home reduced social difficulty scores.	Interview findings not reported, not clear what findings are from family members
Huttmann SE, Windisch W, Storre JH. (2018) Invasive home mechanical ventilation: living conditions and health-related quality of life. [17]	Germany	Cross-sectional study using the QoL in people with COPD and people with neuromuscular disorders (NMD) including MND	n = 32 patients COPD (n=18) NMD (n=14), of which MND (n=6)	5 out of 6 participants with MND recorded QoL scores in the top half of the overall distribution.<br>Living in a private home compared to living in nursing facilities did not influence the scores.	Very wide variation in QoL scores. Only 6 out of 32 participants had MND
Kaub-Wittemer, D., et al. (2003). Quality of life and psychosocial issues in ventilated patients with amyotrophic lateral sclerosis and their caregivers. [34]	Germany	Cross section survey of people on ventilation. Multiple item scale for patients and bespoke	n=52 pairs of plwMND and family members (n=21/52 on TV)	No difference in QOL between NIV and TV<br>Very high percentage of patients would choose TV again but less of carers. And only half of caregivers would choose it for themselves. Family members lower QoL than plwMND.	PlwMND and family members from the same household were asked to complete questionnaires independently, however, it is not known whether this was achieved.

		questionnaire for family members sent to all MND patients on ventilation register		60% of caregivers stopped working due to MND (16% in NV group)	All plwMND were eligible for state-funded care (not available in other contexts)
Kim, C. H., & Kim, M. S. (2014). Ventilator use, respiratory problems, and caregiver well-being in Korean patients with amyotrophic lateral sclerosis receiving home-based care. [39]	Korea	Bespoke questionnaires administered in homes	MND patients (n=141) and caregivers (n=83)	TV associated with higher levels of dyspnoea and additional hospital admissions than NIV, which increased reported caregiver burden;<br>NIV group had more fatigue than TV	56/197 plwMND and 18/101 caregivers excluded (mostly refusals)
Kuzma-Kozakiewicz, M., et al. (2019). An observational study on quality of life and preferences to sustain life in locked-in state. [33]	Poland	Cross sectional study using multiple measures	PlwMND in a locked in state (n=19 - 17/19 participants had TV)	Physical function not linked to subjective wellbeing.<br>All participants would choose the same interventions again.<br>The study found a tendency for caregivers to underestimate wellbeing of plwMND in locked in state and overestimate depressiveness.<br>Suggests psychosocial adaptation in plwMND LIS is good	Small and selective sample. Sample is 18% of all plwMND in a locked in state known to the Polish organisation.
Lulé D, Zickler C, Häcker S, et al. (2009). Life can be worth living in locked-in syndrome. [26]	Germany	Review of literature. No methods described.	Patients with eye movement communication (n=30, n=13 on ventilation) MND and other	Self-reported QoL of locked in state. Patients with higher physical restrictions have better QoL than those less impaired. Also discusses unpublished data on 30 patients and same inverse relationship of function and QoL demonstrated.	No separation of TV and NIV and unclear how many with MND were on ventilation.
Lulé, D., Nonnenmacher, S., Sorg, S. et al. (2014). Live and let die: existential	Germany	Prospective study.	plwMND (n=93)	Suggests that QoL stays stable and not a predictor of the outcomes of ‘vital decisions’.	Significant loses at follow-up.

decision processes in a fatal disease. [29]		Multiple measures used in structured interviews		Suggests that the feeling of being burden was a predictor for plwMND deciding against life sustaining treatments.	Interpretation of QoL may mean study is not directly comparable to other QoL studies.
Peseschkian, T., et al. (2021). A nation-wide, multi-center study on the quality of life of ALS patients in Germany. [32]	Germany	Cross sectional questionnaire survey	Attendees at 17 MND centres across Germany (n=325, 4.3% using TV)	TV had positive effect on QoL<br><br>Useful to see QoL of other MND patients at various stages/ technologies for comparison	Only a small part of paper. Limitations of those motivated to complete the questionnaire.
Rabkin, J. et al. (2006). Predictors and course of elective long-term mechanical ventilation: A prospective study of ALS patients. [30]	USA	Prospective cohort study with some longitudinal follow-up over 4 years. Multiple measures used	plwMND (n=72, n=14 with TV)<br>Family members (n=11) interviewed with plwMND	Those choosing TV were more likely to be younger, have children under 21, higher education and household income.<br>Some decrease in QoL after average of 33 months on TV.<br>Caregivers for TV reported substantial emotional burden but retained overall satisfaction with caregiving;<br>14% at baseline versus 50% of TV patients at death reported to have cognitive problems, a few moderate/severe	14/72 chose TV and 11 followed-up (58 died by end of study).<br>Cognitive assessments limited
Rivara, M., et al. (2016). Management of the patient with advanced amyotrophic lateral sclerosis: The caregiver's opinion. [44]	Italy	Bespoke caregiver/family member survey	Family members (n=70, TV n=24 compared with NIV n=46)	Carers reporting ‘burn out’, and ‘difficulty’ higher in carers caring for plwMND with TV<br>Burnout 45.8% as against 21.7%<br>Difficulty 58.3% as against 26.1%	Only top-level data is segregated by NIV and TV
Rousseau, M. et al. (2011). Quality of life of ALS and LIS patients with and without invasive mechanical ventilation. [35]	France	Cross-sectional study using multiple measures	Compared n=12 patients with TV (n=8 MND, n=4 LIS) to QoL of n=22 patients without TV	QoL was not significantly different between TV and not TV patients.	MND results not reported separately.<br>No info on how tests were administered and completed, given functional status of participants.

			(n=19 MND, n=3 LIS)		
Sutherland, J. (2020). A decision to live. [37]	USA	Personal narrative	plwMND (n=1)	A personal narrative from a person with MND about their choice to undergo TV. Key elements of the decision were based on the level of burden on his wife and adult children, his lack of suffering, physically or mentally versus a desire to live. Feeling he was still able to contribute and maintain dignity. Their financial position. Framing as choice to live by having the TV, or not live and wait to die.	Only one person's experience. Limited detail on living with TV, more emphasis on the decision-making process.
Tülek Z, Özakgül A, Alankaya N, Dik A, Kaya A, Ünalan PC, Özaydin AN & Idrisoğlu HA (2023) Care burden and related factors among informal caregivers of patients with amyotrophic lateral sclerosis. [40]	Turkey	Descriptive, mixed methods study including interviews with MND patients and family caregivers.	MND patient-family member dyads (n=108, n=56 TV)	Caregiver burden was related to gender and the functional state of the patient, as well as relation to the patient, sex, health status, time spent on care, social support and living in the same house with a limited environment. Walking ability, PEG, tracheostomy, and communication problems <u>not</u> associated with carer burden. Attributed to greater use of paid care.	plwMND not exclusively TV users. No measure of cognitive functioning or use of paid care.
Veronese, S., et al. (2014). The last months of life of people with amyotrophic lateral sclerosis in mechanical invasive ventilation: A qualitative study. [38]	Italy	Qualitative – semi-structured interviews	Bereaved family members of pt with TV (n=19)	Those with paid help, less likely to report reduced QoL. QoL over all reduced but satisfied. Families noted pressure from doctors to have the intervention. Advance directives not legally binding and can be overridden by attending clinicians.	PlwMND views presented by proxy
Vianello, A., et al. (2011). Survival and quality of life after tracheostomy for acute respiratory failure in patients	Italy	Retrospective cohort study over 13 years	PlwMND with TV (n=60)	Mean interval from diagnosis to TV was 2 years (SD 20 months),<br>All patients were discharged safely home after TV. In sub-group QoL study mean 20 months after TV n=11/13 reported positive	Only 13/60 still alive for interview component and contribution to QoL information.

with amyotrophic lateral sclerosis. [31]				view and would make same decision again; 2/13 were severely depressed<br>No difference in QoL score or demographics in those choosing TV compared to a historical group not receiving TV	
Winther, D., Kirkegaard Lorenzen, C., & Dreyer, P. (2020). Everyday life experiences of close relatives of people with amyotrophic lateral sclerosis receiving home mechanical ventilation - A qualitative study. [43]	Denmark	Qualitative interview study with a phenomenological hermeneutic approach.	Family members of plwMND (n=11, TV n=9)	Four main themes:<br>Family members as caregivers and care co-ordinators. Formal caregivers as essential, but also a burden. Living on permanent standby with limited personal space. Family members interpret their role as maintaining QoL for plwMND. Living with TV is a joint enterprise (use of 'we')	NIV/TV findings not reported separately.<br>3 family members belonged to a support group and were interviewed together.
